# Circadian disruption by short light exposure and a high energy diet impairs glucose tolerance and increases cardiac fibrosis in *Psammomys obesus*

**DOI:** 10.1038/s41598-021-89191-7

**Published:** 2021-05-06

**Authors:** Victoria A. Nankivell, Joanne T. M. Tan, Laura A. Wilsdon, Kaitlin R. Morrison, Carmel Bilu, Peter J. Psaltis, Paul Zimmet, Noga Kronfeld-Schor, Stephen J. Nicholls, Christina A. Bursill, Alex Brown

**Affiliations:** 1 South Australian Health & Medical Research Institute, Adelaide, Australia; 2 Faculty of Health and Medical Science, The University of Adelaide, Adelaide, Australia; 3 School of Zoology, Tel Aviv University, Tel Aviv, Israel; 4 Department of Diabetes, Monash University, Melbourne, Australia; 5 Monash Cardiovascular Research Centre, Victorian Heart Institute, Monash University, Melbourne, Australia; 6 ARC Centre of Excellence for Nanoscale BioPhotonics, The University of Adelaide, Adelaide, Australia; 7 Vascular Research Centre, Lifelong Health Theme, South Australian Health & Medical Research Institute, North Terrace, Adelaide, SA 5000 Australia

**Keywords:** Experimental models of disease, Preclinical research

## Abstract

Type 2 diabetes mellitus (T2DM) increases cardiac inflammation which promotes the development of cardiac fibrosis. We sought to determine the impact of circadian disruption on the induction of hyperglycaemia, inflammation and cardiac fibrosis. Methods: *Psammomys obesus* (*P. obesus*) were exposed to neutral (12 h light:12 h dark) or short (5 h light:19 h dark) photoperiods and fed a low energy (LE) or high energy (HE) diet for 8 or 20 weeks. To determine daily rhythmicity, *P. obesus* were euthanised at 2, 8, 14, and 20 h after ‘lights on’. Results: *P. obesus* exposed to a short photoperiod for 8 and 20 weeks had impaired glucose tolerance following oral glucose tolerance testing, compared to a neutral photoperiod exposure. This occurred with both LE and HE diets but was more pronounced with the HE diet. Short photoperiod exposure also increased myocardial perivascular fibrosis after 20 weeks on LE (51%, *P* < 0.05) and HE (44%, *P* < 0.05) diets, when compared to groups with neutral photoperiod exposure. Short photoperiod exposure caused elevations in mRNA levels of hypertrophy gene *Nppa* (atrial natriuretic peptide) and hypertrophy transcription factors *Gata4* and *Mef2c* in myocardial tissue after 8 weeks. Conclusion: Exposure to a short photoperiod causes impaired glucose tolerance in *P. obesus* that is exacerbated with HE diet and is accompanied by an induction in myocardial perivascular fibrosis.

## Introduction

Cardiovascular disease is the leading cause of death worldwide^1^. Type 2 Diabetes Mellitus (T2DM) is regarded as pan epidemic, with more than 640 million people predicted to have T2DM by 2040^2^. It is well-established that there is a link between cardiovascular disease (CVD) and T2DM with them frequently occurring simultaneously along with an associated elevated risk of adverse outcomes^1,3,4^. Specifically, people with diabetes or pre-diabetes are more likely to develop cardiovascular disease than their non-diabetic counterparts^1,3,4^. Diabetes causes a range of myocardial abnormalities that can lead to heart failure including left ventricular hypertrophy, an impaired tolerance to ischemic injury and pathophysiological changes in the myocardium that can result in diabetic cardiomyopathy^5–9^. The characteristics of diabetic cardiomyopathy include: myocardial fibrosis, dysfunctional remodelling, diastolic and systolic dysfunction, an elevation in collagen-based myocytes and extracellular matrix stiffness and impaired calcium handling. Inflammation is increasingly recognised as a central underlying cause of these changes which can lead to heart failure^5,10^.

The circadian system is important for the regulation and maintenance of metabolic pathways^11,12^. Regulated by the central hypothalamic clock in the supra-chiasmatic nucleus (SCN) and peripheral clocks in almost every cell of the body, metabolic pathways cycle within a periodic 24-h light/dark environment^13^. The circadian clock is synchronised with solar time by exposure to light. However with the Industrial Revolution and the invention of electric lighting the natural predictable pattern of light and darkness has been disturbed^14^. Furthermore, the increasing amount of shift work now undertaken exposes employees to additional disruptions in the natural daily cycle, with them experiencing very much reduced light throughout a 24-h period^15^. Disruptions to the circadian system have been shown to have a range of negative health impacts including increased inflammation, impaired glucose regulation, elevated risks of obesity, cardiovascular disease, metabolic syndrome, and T2DM^13–19^.

Animal studies of T2DM and circadian rhythms are difficult to simulate in nocturnal rodents^20^. However, a unique rodent model, the gerbil *Psammomys obesus (P. obesus)*, is diurnal and spontaneously develops diet induced obesity and T2DM when fed a typical laboratory rodent diet^21–23^. This has significant advantages over the streptozotocin infusion model that causes destruction of pancreatic beta cells and therefore more closely mimics Type 1 DM^20^. *P. obesus* have evolved in their native environment, where they feed on a diet of low energy saltbush on which they remain lean and normoglycemic. When held under laboratory conditions and fed a standard rodent diet with a higher energy content, T2DM develops spontaneously^23,24^. This is accompanied by weight gain and cardiac hypertrophy^21,22^. The onset of these pathophysiological consequences can be accelerated with exposure to circadian disruption^22,25^. Taken together, using shortened light exposure (5 h light:19 h dark) interventions, in parallel with a high energy diet, makes the diurnal *P. obesus* a unique and ideal model in which to model the effects of shift work on T2DM-induced cardiovascular complications.

Accordingly, this study sought to determine the effect that a typical “westernised” shift worker lifestyle with high energy diet and disrupted circadian cycle on the development of T2DM, cardiac fibrosis and inflammation. Using the unique pathophysiologically relevant *P. obesus* model, we simulated this scenario using a rodent chow and exposure to a short photoperiod for either 8 or 20 weeks.

## Results

### Effect of high energy diet and shorter photoperiod on weights and glucose levels

**Figure 1:**
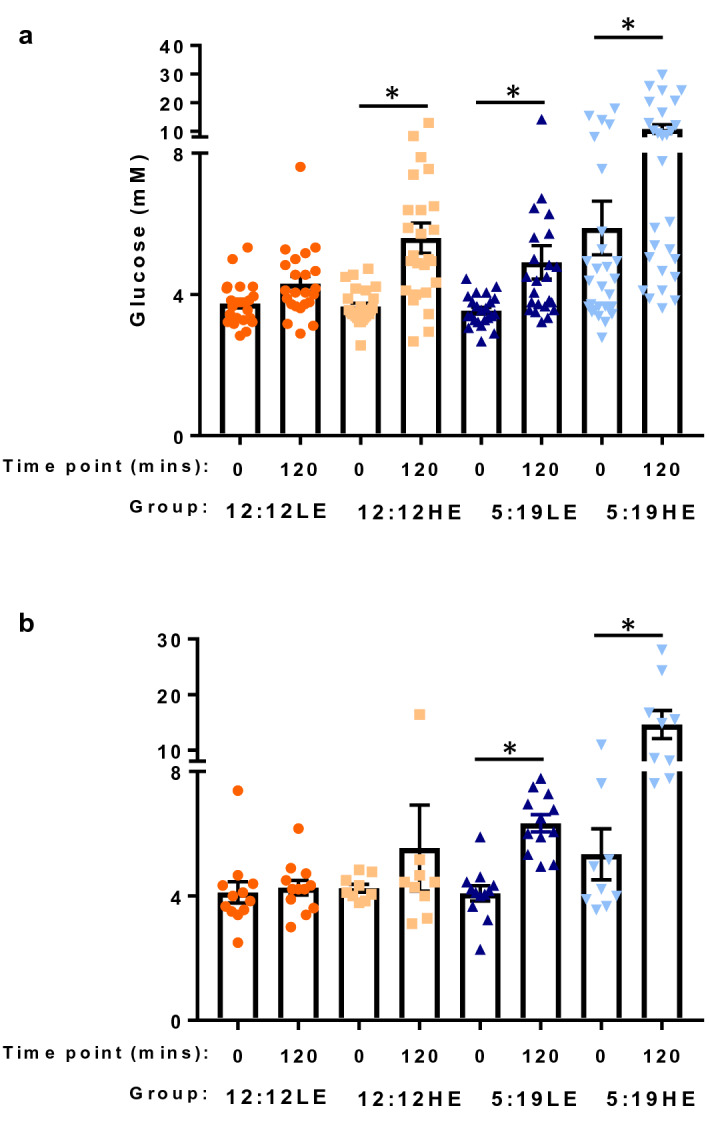
High energy diet and Short photoperiod cause abnormal glucose tolerance. Oral glucose tolerance tests (OGTT) were performed in *P. obesus* were exposed to low energy (LE) or high energy (HE) diet and either a neutral photoperiod (12 light:12 dark) or short photoperiod (5 light:19 dark) for 8 (**a**) or 20 weeks (**b**). *P. obesus* were fasted for 4 h. A dose of 2 g glucose/kg body weight was administered via gavage. Blood was collected at time 0 and 120 min later. Mean ± SEM, n = 6–15/group. *P < 0.05 compared to time point 0.

*P. obesus* fed the HE diet and exposed to the short photoperiod (5:19HE group) for 8 weeks had significantly higher heart and body weights and a lower heart:body weight ratio, compared to the 12:12LE control group (Table 1). After 8 weeks, fasting blood glucose levels were elevated in the group exposed to a short photoperiod and HE diet (5:19HE), compared to all other groups (12:12LE 53%; 12:12HE 79%; 5:19LE 85%, Table 1). Glucose tolerance was impaired in groups that were exposed to a HE diet or short photoperiod or both (12:12HE, 5:19LE and 5:19HE groups). This was demonstrated by a significant elevation in glucose levels 120 min after receiving a bolus infusion of glucose, when compared to the corresponding 0 min timepoints (Fig. 1a, P < 0.05 for all).

**Table 1:** Weights and glucose levels of *P. obesus* exposed to neutral or short photoperiods and a high energy or low energy diet for 8 weeks.

8-week cohort	12:12LE	12:12HE	5:19LE	5:19HE
Heart weight (g)	0.55 ± 0.02	0.60 ± 0.03	0.57 ± 0.03	0.61 ± 0.02^†^
Body weight (g)	200.90 ± 8.14	249.00 ± 13.70	237.5 ± 12.34^†^	249.50 ± 5.74^††^
Heart:body weight ratio (× 10^–4^)	27.41 ± 1.36	24.51 ± 1.26	24.34 ± 1.05	24.31 ± 0.63^†^
Fasting glucose levels (mM)	3.74 ± 0.13	3.65 ± 0.10	3.54 ± 0.09	5.88 ± 0.76^†#,^^

*P. obesus* were exposed to low energy (LE) or high energy (HE) diet and either a neutral photoperiod (12 light:12 dark) or short photoperiod (5 light:19 dark) for 8 weeks. Data presented as Mean ± SEM.

^†^*P* < 0.05, ^††^*P* < 0.01 vs. 12:12LE; ^#^*P* < 0.05, vs. 12:12HE; ^*P* < 0.05 vs. 5:19LE.

In the 20-week cohort, there were no significant differences in heart weight across the four groups (Table 2). However, the 5:19HE group gained significantly more body weight compared to both the 12:12LE and 5:19LE groups. No differences in heart:body weight ratios were observed. After 20 weeks, there were no changes in fasting blood glucose levels (Table 2). Glucose tolerance was, however, impaired in groups that were exposed to a short photoperiod (5:19LE and 5:19HE groups) as glucose levels were significantly elevated 120 min after glucose infusions, compared to corresponding 0 min timepoints (Fig. 1b, P < 0.05 for all).

**Table 2:** Weights and glucose levels of *P. obesus* exposed to neutral or short photoperiods and a high energy or low energy diet for 20 weeks.

20-week cohort	12:12LE	12:12HE	5:19LE	5:19HE
Heart weight (g)	0.71 ± 0.02	0.60 ± 0.03	0.57 ± 0.03	0.61 ± 0.02
Body weight (g)	221.30 ± 9.37	239.70 ± 14.05	218.00 ± 9.02	256.70 ± 6.08^††,^^
Heart:body weight ratio (× 10^–4^)	32.62 ± 1.56	30.86 ± 2.17	32.46 ± 1.94	30.05 ± 1.36
Fasting glucose levels (mM)	4.11 ± 0.34	4.24 ± 0.13	4.08 ± 0.24	5.34 ± 0.82

*P. obesus* were exposed to low energy (LE) or high energy (HE) diet and either a neutral photoperiod (12 light:12 dark) or short photoperiod (5 light:19 dark) for 20 weeks. Data presented as Mean ± SEM.

^††^*P* < 0.01 vs. 12:12LE; ^#^*P* < 0.05 vs. 12:12HE; ^*P* < 0.05 vs. 5:19LE.

### 20 weeks of high energy diet and shorter photoperiod induced perivascular fibrosis in heart tissue

**Figure 2:**
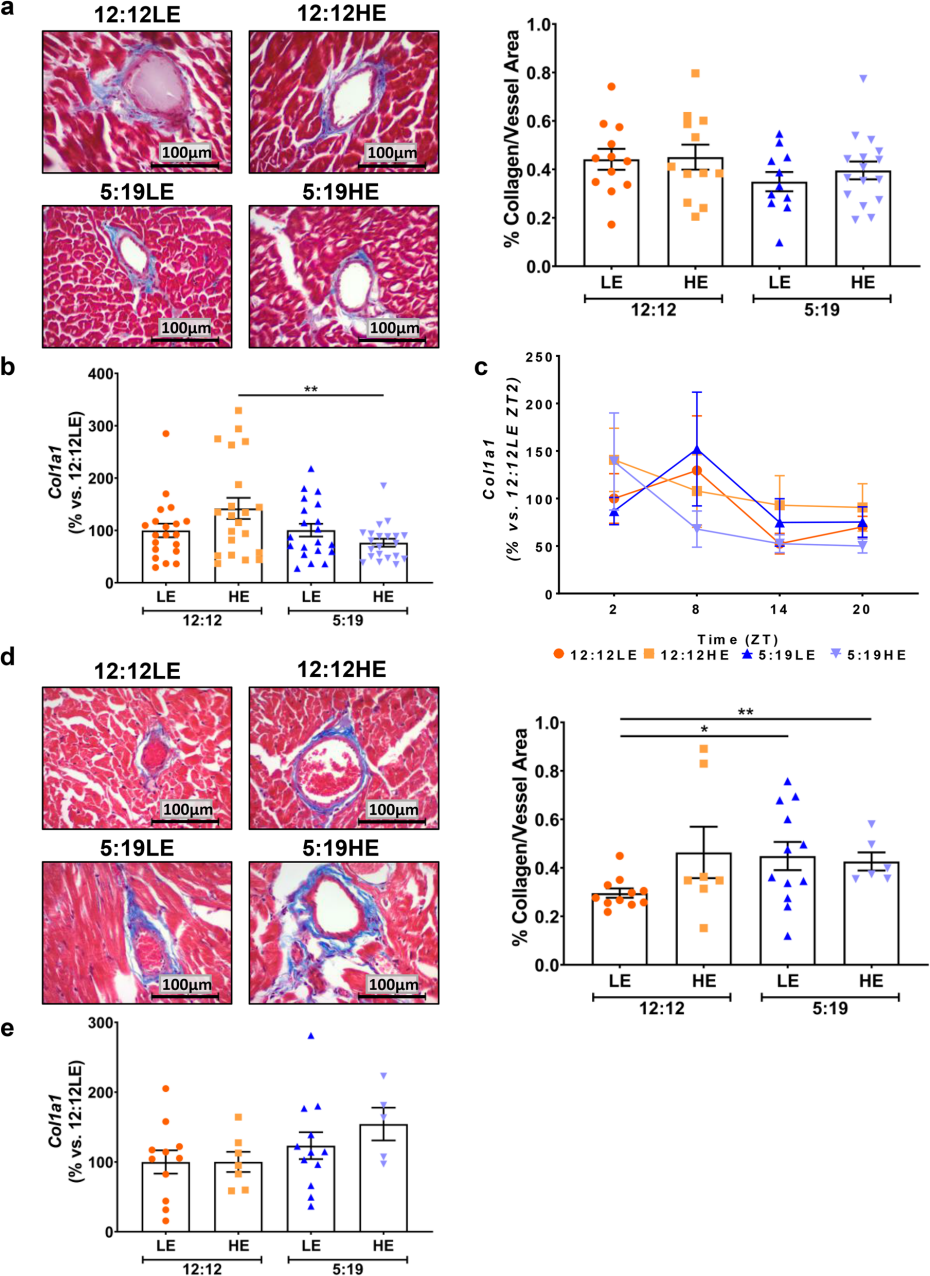
20 weeks of high energy diet and short photoperiod increases myocardial perivascular fibrosis. *P. obesus* were exposed to low energy (LE) or high energy (HE) diet and either a neutral photoperiod (12 light:12 dark) or short photoperiod (5 light:19 dark) for 8 or 20 weeks. (**a**) Representative images of Masson’s trichrome-stained hearts depicting collagen deposition surrounding the vessels after 8-weeks of treatment (n = 11–15/group). Perivascular fibrosis was analysed by determining area of blue staining around selected vessels and normalised to the vessel area. (**b**) Average *Col1a1* gene expression normalised using the ^ΔΔ^*Ct* method to *Cyclophilin* and to the 12:12LE group. (**c**) Daily *Col1a1* rhythms at hourly time points post-‘lights on’. (**d**) Representative images of perivascular fibrosis in myocardial sections after 20 weeks of treatment with analyses (n = 6–12/group). (**e**) Average *Col1a1* gene expression. Mean ± SEM. *P < 0.05, **P < 0.01.

Perivascular fibrosis was detected by staining for the presence of collagen around the heart vessels. At 8 weeks, there were no changes in the amount of collagen deposition (Fig. 2a), either in the animals fed a HE diet, nor exposed to a short photoperiod. Animals on both the HE diet and the short photoperiod (5:19HE) had reduced expression of the collagen gene *Col1a1* (46%, *P* < 0.05), when compared to the 12:12HE group (Fig. 2b). However, no daily rhythm differences were observed between time points across all the groups (Fig. 2c).

In the 20-week cohort, when compared to the 12:12LE group, there was a significant increase perivascular fibrosis with higher levels of collagen deposition around myocardial vessels in both groups of animals exposed to a short photoperiod (Fig. 2d, 5:19LE 51%; 5:19HE 44%). There was a trend for an increase in myocardial *Col1a1* expression in the 5:19HE group (54%, P = 0.06), when compared to 12:12HE animals (Fig. 2e). Overall, exposure of a short photoperiod for 20 weeks appears to be the main effector stimulating myocardial perivascular fibrosis.

### Effects of high energy diet and short photoperiod on hypertrophy genes expression and cardiomyocyte size

**Figure 3:**
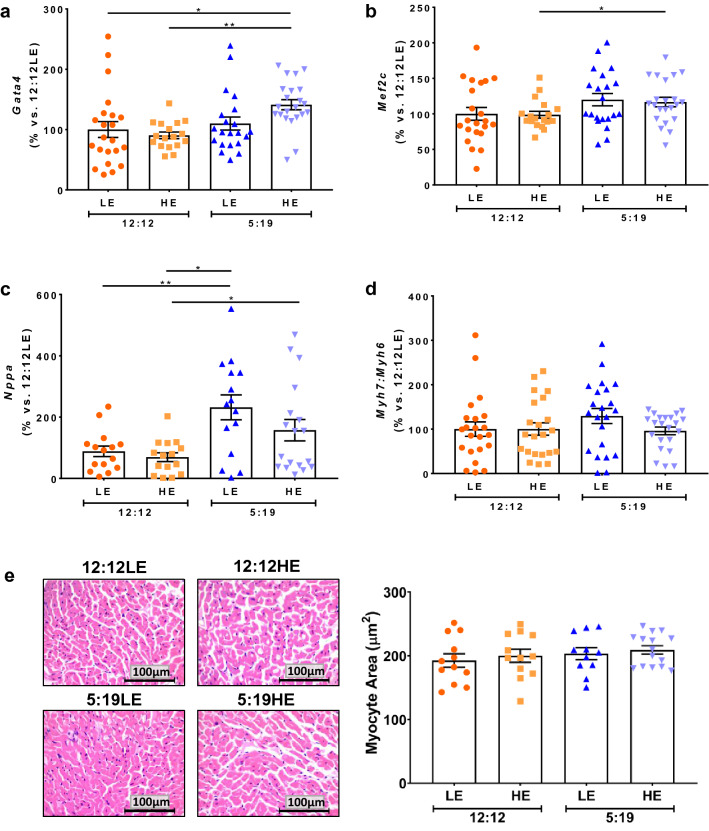
8 Weeks of High energy diet and shorter photoperiod increase hypertrophy gene expression. *P. obesus* were exposed to low energy (LE) or high energy (HE) diet and either a neutral photoperiod (12 light:12 dark) or short photoperiod (5 light:19 dark) for 8 or 20 weeks. Cardiac hypertrophy genes were measured by qPCR including: (**a**) *Gata4*, (**b**) *Merf2c*, (**c**) *Nppa* and (**d**) the *Myh7*:*Myh6* ratio. (**e**) Representative images of H&E-stained hearts after 8 weeks of treatment. Myocyte size (area) was analysed by determining the area of eosin staining divided by the number of nuclei/image. Mean ± SEM, n = 11–15/group. *P < 0.05, **P < 0.01, ***P < 0.001.

We next assessed the hypertrophy markers atrial natriuretic peptide (ANP) and myosin heavy chain (MHC)α (adult isoform) and MHCβ (foetal isoform), represented by genes *Nppa*, *Myh6* and *Myh7*, respectively. We also measured changes mRNA levels of the transcription factors that control these genes, *Gata4* and *Mef2c*^26^. Morphological changes in cardiomyocyte size, representing hypertrophy, were also determined. In the 8-week cohort, we found that there was a significant increase in *Gata4* in hearts of the 5:19HE group, when compared to 12:12LE and the 12:12HE groups (Fig. 3a, *P* < 0.05). *Mef2c* was also elevated in the 5:19HE group, compared to the 12:12LE (Fig. 3b, *P* < 0.05). Demonstrating that a combination of a short photoperiod and/or HE diet increases these hypertrophy transcription factors. Significant increases were also noted in *Nppa* (i.e. ANP) in both groups exposed to a short photoperiod such that *Nppa* was higher in the 5:19LE and 5:19HE groups, when compared to the 12:12HE group (Fig. 3c, *P* < 0.05). This shows that short photoperiod exposure is the main driver of these changes. No changes occurred in the *Myh7*:*Myh6* ratio, a marker of cardiac function, between groups (Fig. 3d, *P* < 0.05). Despite the overall elevation in hypertrophy-related genes in the *P. obesus* exposed to HE and short photoperiod (5:19HE), there were no changes in cardiomyocyte size across all groups in the 8-week cohort (Fig. 3e).

**Figure 4:**
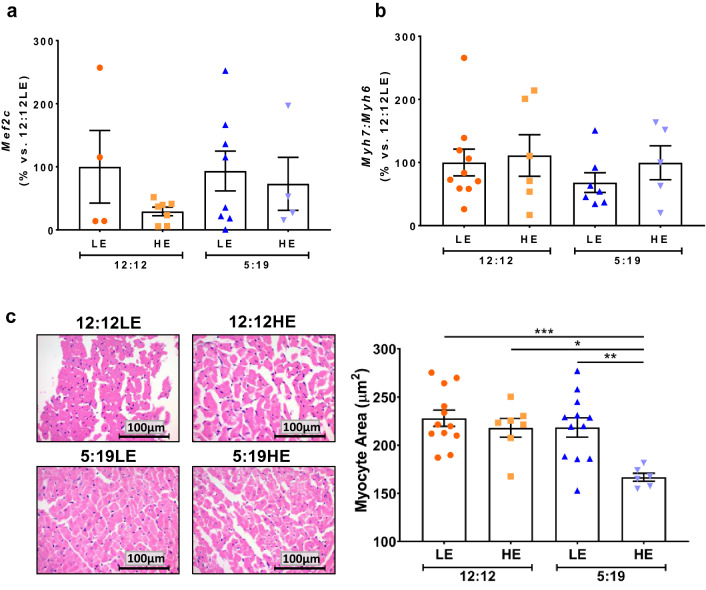
20 weeks of high energy diet and shorter photoperiod reduce cardiomyocyte size. *P. obesus* were exposed to low energy (LE) or high energy (HE) diet and either a neutral photoperiod (12 light:12 dark) or short photoperiod (5 light:19 dark) for 8 or 20 weeks. Cardiac hypertrophy genes were measured by qPCR including: (**a**) *Merf2c* and (**b**) the *Myh7*:*Myh6* ratio. (**c**) Representative images of H&E-stained hearts after 20 weeks of treatment. Myocyte size (area) was analysed by determining the area of eosin staining divided by the number of nuclei/image. Mean ± SEM, n = 6–12/group. *P < 0.05, **P < 0.01, ***P < 0.001.

In the 20-week cohort, no changes were found in *Merf2C* or the *Myh7*:*Myh6* ratio between groups (Fig. 4a,b, *P* < 0.05). However, histological analyses revealed that *P. obesus* exposed to the high energy diet and short photoperiod (5:19HE) had significantly smaller cardiomyocytes, when compared to all other groups (Fig. 4c, *P* < 0.05).

### Expression of Clock, inflammatory and pro-fibrotic genes after 8 weeks of high energy diet and/or short photoperiod

**Figure 5:**
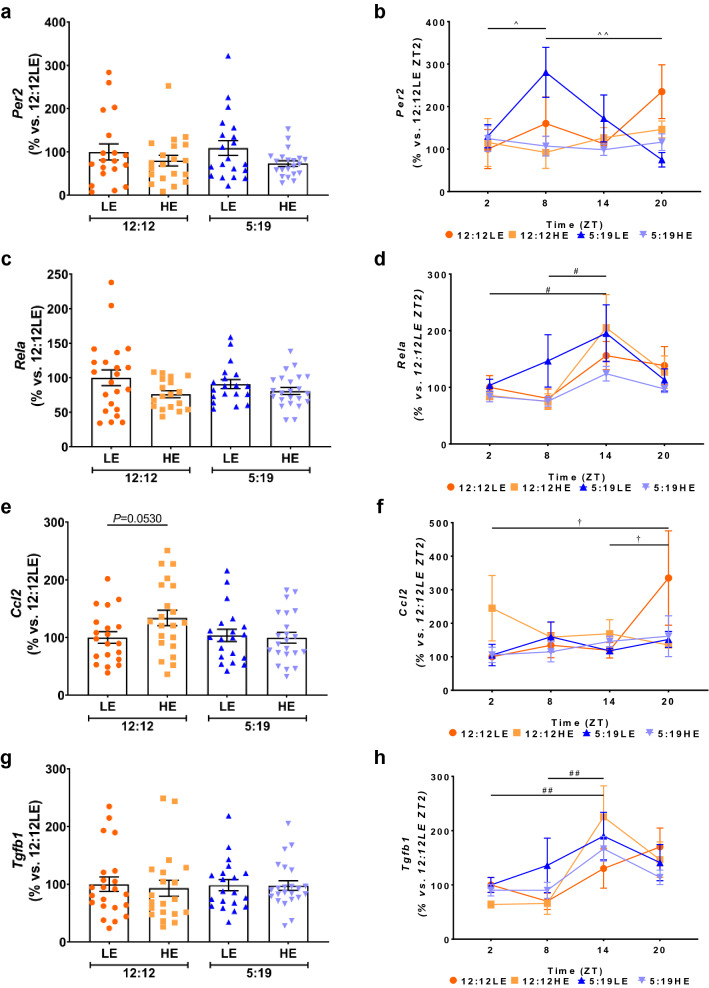
Expression of *Per2* and inflammatory markers after 8 weeks of high energy diet and/or shorter photoperiod. *P. obesus* were exposed to low energy (LE) or high energy (HE) diet and either a neutral photoperiod (12 light:12 dark) or short photoperiod (5 light:19 dark) for 8 weeks. Expression of (**a**,**b**) *Per2*, (**c**,**d**) *Rela*, (**e**,**f**) *Ccl2* and (**g**,**h**) *Tgfb1* genes in the myocardial tissues. Average gene expression was determined across the 4 groups and normalised using the ^ΔΔ^*Ct* method to *Cyclophilin* and to the 12:12LE group (left panels). Daily rhythms were determined by measuring gene expression at hourly time points post-lights on. Mean ± SEM, n = 11–15/group. ^†^*P* < 0.05 within 12:12LE group; ^#^*P* < 0.05, ^##^*P* < 0.01 within 12:12HE group; ^^^*P* < 0.05, ^^^^*P* < 0.01, within 5:19LE group.

We next assessed the average expression and daily rhythms of the established clock gene *Per2*, inflammatory genes *Rela* and *Ccl2,* and pro-fibrotic gene *Tgfb1* in our 8-week cohort. No significant differences were observed in the average expression of *Per2* across all four groups irrespective of diet or photoperiod (Fig. 5a). It has been shown that the expression of *Per2* will change over a photoperiod^27^. As such, we measured the gene expression changes across a 24-h period. Daily *Per2* rhythms changed in animals exposed to short photoperiod fed a LE diet (5:19LE) (Fig. 5b). In this cohort, *Per2* expression was elevated at ZT8 when compared to ZT2 and ZT20.

Assessment of inflammatory genes showed that while the average *Rela* (*p65-NF-κB*) mRNA levels did not differ across the four groups (Fig. 5c), the daily *Rela* rhythm was found to increase at ZT14 compared to ZT2 and ZT8 in the 12:12HE group (Fig. 5d), indicating a cyclic effect over the day with the peak at ZT14 with a HE diet but normal photoperiod. High energy diet alone with a normo-photoperiod (12:12HE) caused a trend for an increase in the average myocardial *Ccl2* expression in the 12:12HE group, when compared to the 12:12LE group (Fig. 5e, 34%, *P* = 0.0530). Daily *Ccl2* rhythm was significantly different in the 12:12LE group with elevated *Ccl2* levels observed at ZT20 compared to ZT2 and ZT14 (Fig. 5f), indicating that *Ccl2* gene expression increases after a longer light exposure in our control group (12:12LE). By contrast, the daily *Ccl2* rhythms in the groups exposed to HE diet and/or short photoperiod (12:12HE, 5:19LE or 5:19HE) were not altered. While no differences were observed with average *Tgfb1* mRNA levels (Fig. 5g), variations in daily rhythm were observed in the 12:12HE group (Fig. 5h). Similar to the *Rela* patterns, *Tgfb1* mRNA levels in the 12:12HE group increased at ZT14 compared to ZT2 and ZT8, indicating a cyclic effect over the day with the peak at ZT14. This was stimulated by a HE diet but normal photoperiod.

### Expression of apoptosis genes and Serca2 after 8 weeks of high energy diet and/or short photoperiod

**Figure 6:**
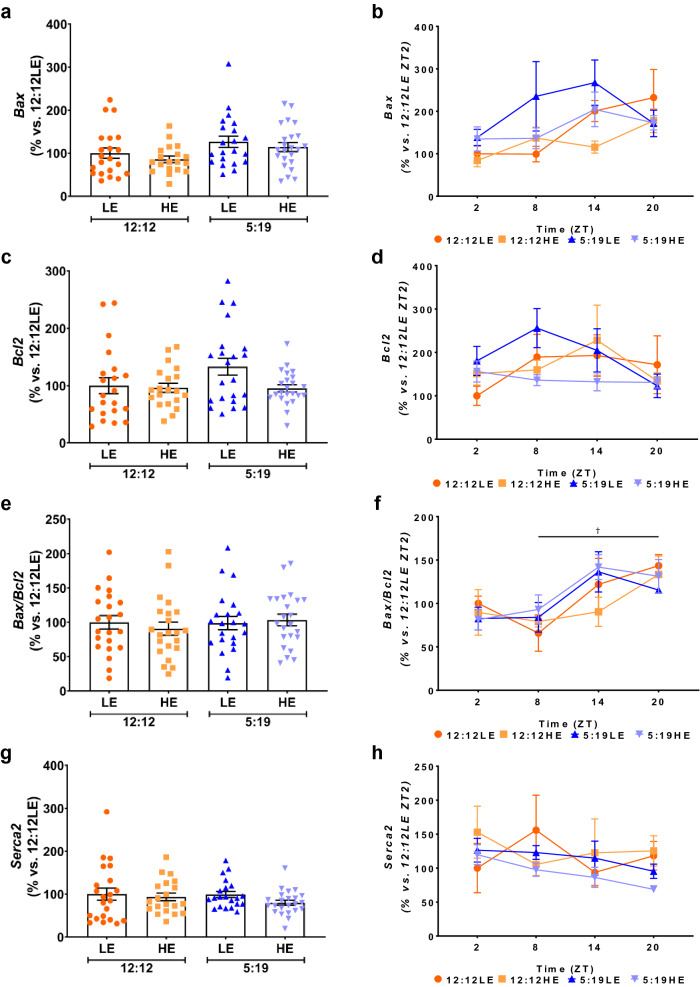
Expression of apoptosis markers and Serca2 after 8 weeks of high or low energy diet and/or shorter photoperiod. *P. obesus* were exposed to low energy (LE) or high energy (HE) diet and either a neutral photoperiod (12 light:12 dark) or short photoperiod (5 light:19 dark) for 8 weeks. Expression of (**a**,**b**) *Bax*, (**c**,**d**) *Bcl2*, (**e**,**f**) *Bax/Bcl2* and (**g**,**h**) *Serca2* in the 8-week cohort. Average gene expression was determined and normalised using the ^ΔΔ^*Ct* method to *Cyclophilin* and to the 12:12LE group (n = 11–15/group, left panels). Daily rhythms were determined by measuring gene expression at hourly time points post-lights on (n = 2–6/group, right panels). Mean ± SEM ^†^*P* < 0.05 within 12:12LE group.

We then determined whether high energy diet with a short photoperiod affected apoptosis by measuring the expression of *Bax*, a pro-apoptotic gene in tandem with the anti-apoptotic gene *Bcl2*. Overall, there were no differences in either the average expression or daily rhythms of *Bax* and *Bcl2* across all four groups (Fig. 6a–d). The average *Bax/Bcl2* ratio, a marker of apoptosis, showed no differences between groups (Fig. 6e). However, there was a difference in its daily rhythm in the 12:12LE group (Fig. 6f). The *Bax/Bcl2* ratio increased at ZT20 compared to ZT8, indicating a cyclic effect of overall apoptosis that increases over a longer photoperiod. No differences were observed in either the average expression or daily rhythm of *Serca2*, a heart contractility gene (Fig. 6g,h).

### Effect of 20 weeks of high energy diet and/or shorter photoperiod on gene expression in sand rat hearts

**Figure 7:**
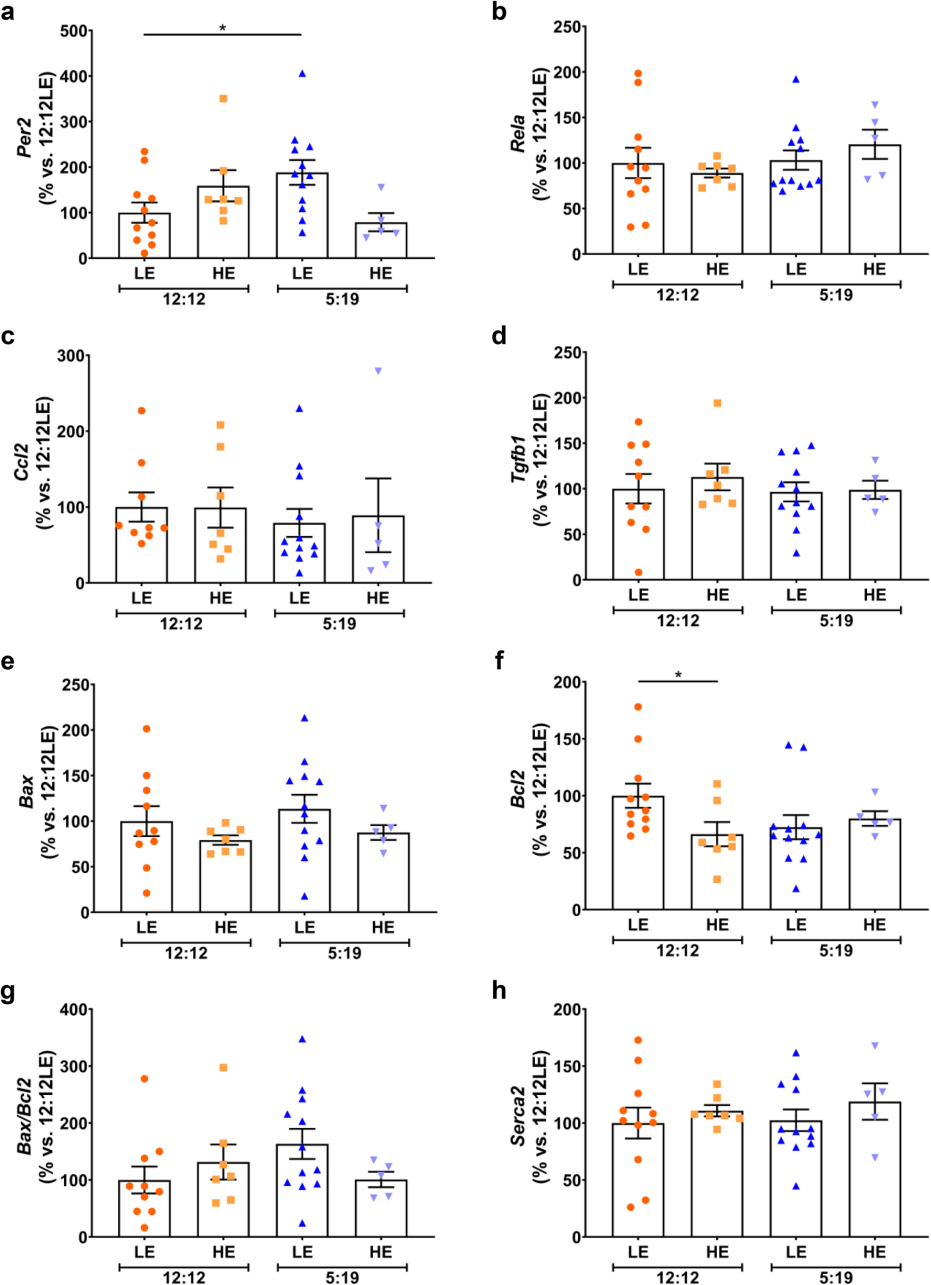
Effect of 20 Weeks of low or high energy diet and/or shorter photoperiod on gene expression. *P. obesus* were exposed to low energy (LE) or high energy (HE) diet and either a neutral photoperiod (12 light:12 dark) or short photoperiod (5 light:19 dark) for 20 weeks. Average gene expression of (**a**) *Per2*, (**b**) *Rela*, (**c**) *Ccl2*, (**d**) *Tgfb1*, (**e**) *Bax*, (**f**) *Bcl2*, (**g**) *Bax/Bcl2* and (**h**) *Serca2* was determined across the 4 groups and normalised using the ^ΔΔ^*Ct* method to *Cyclophilin* and to the 12:12LE group of the 20-week cohort. Mean ± SEM, n = 6–12/group. *P < 0.05.

Whether HE diet and/or a short photoperiod affected apoptotic and contractility genes after 20-weeks of exposure was then assessed. *Per2* mRNA levels were significantly higher in the 5:19LE group when compared to the 12:12LE (Fig. 7a, 1.2-fold, *P* < 0.05), indicating an effect of short light exposure. No significant differences were observed in the expression of inflammatory genes *Rela* (Fig. 7b) and *Ccl2* (Fig. 7c), pro-fibrotic gene *Tgfb1* (Fig. 7d), or the pro-apoptotic gene *Bax* (Fig. 7e). The 12:12HE animals had significantly lower expression of the anti-apoptotic gene *Bcl2* (Fig. 7f, 35%, *P* < 0.05). There were no changes in the *Bax/Bcl2* ratio (Fig. 7g) or *Serca2* (Fig. 7h) mRNA levels across all four groups.

## Discussion

Circadian disruption caused by disordered exposure to light increases a plethora of metabolic-related complications including T2DM and cardiovascular disease^11^. Using the unique rodent model of *P. obesus* we investigated the effects of circadian disruption on the onset of T2DM and myocardial pathophysiology. After both 8 weeks and 20 weeks of exposure we found that a short photoperiod (5:19) caused abnormal glucose tolerance, when compared to neutral photoperiod (12:12) exposure. This occurred with both LE and HE diets, but was exacerbated with the HE diet which was coupled to a significant increase in body weight. In parallel, we found the short photoperiod induced myocardial perivascular fibrosis after 20 weeks, but not 8 weeks, which occurred on both the LE and HE diets. This corresponded with an increase in *Col1a1* collagen gene expression in the 5:19HE group, compared to the 12:12HE group. Furthermore, we found that the hearts of *P. obesus* in the 5:19HE group displayed increases in the expression of hypertrophy genes *Gata4*, *Merf2c* and *Nppa* after 8 weeks but not 20 weeks. Taken together, by simulating the conditions of human shift work with *P. obesus*, our data imply that shift workers exposed to circadian disruption may be at increased risk of developing T2DM, which is more pronounced with an HE diet. This induces features of cardiac fibrosis, a factor involved in the development of heart failure. The findings suggest that interventions that correct circadian disruption are likely to show benefit.

Hyperglycaemia increases cardiac fibrosis via an induction of pro-inflammatory signals that increase collagen deposition and subsequently become organised into a fibrotic scar^5^. The fibrosis occurs either around myocardial vessels (perivascular fibrosis) or interstitially^28,29^. In the current study, we have shown that when placed on a HE diet for either 8 or 20 weeks *P. obesus* display features of T2DM development. Circadian disruption via a shortened photoperiod substantially increased both fasting glucose levels after 8, but not 20 weeks, of exposure and impaired glucose tolerance as determined using OGTT at both time points. Of particular interest was that in the 20-week group the HE diet alone did not induce impaired glucose tolerance, but only occurred when combined with a short photoperiod. This demonstrates the important regulatory effects of disrupted circadian cycles on the development of T2DM. These findings suggest that exposure to disrupted circadian cycles causes exacerbated detrimental effects on glucose tolerance and weight gain, particularly when accompanied with a HE diet.

Another factor that reflected the exacerbation of abnormal glucose tolerance in the 20-week short photoperiod groups, was the induction of myocardial perivascular fibrosis. This was not present in the 8-week cohorts suggesting that it takes a longer period of time for the fibrotic deposits to develop. Furthermore, in the 20-week group the development of perivascular fibrosis only became evident in the *P. obesus* exposed to the short photoperiod but was independent of diet. Consistent with the increase in myocardial perivascular fibrosis, there was an increase in the expression of myocardial expression of collagen type 1 gene *Col1a1.* These findings support other studies in *P. obesus*^24^ that have reported increases in myocardial perivascular fibrosis and *Col1a1* and has been noted in alternate rodent models of Type 1 and Type 2 diabetes^5,30–32^. However, our study is the first to demonstrate that circadian disruption can drive these pathophysiological changes in the myocardial tissue, independent of diet.

Increases in myocardial perivascular fibrosis and *Col1a1* were not accompanied by increases in inflammation in the 20-week groups with a short photoperiod. Proinflammatory genes *CCl2* and *Rela* remained unchanged but are reported to be the underlying mechanistic drivers of hyperglycaemic-driven myocardial fibrosis^6^. Furthermore, pro-fibrotic gene *TGF-B1* that is also anti-inflammatory did not change in the 20-week group. A likely explanation for the lack of change is that after 20-weeks the earlier inflammatory phase had already resolved and the subsequent proliferative phase of repair, involving *TGF-B1* (which proceeds collagen deposition^33^), was also complete. At the 8-week time point some of our data support this concept. In the earlier 8-week 12:12HE group, myocardial *CCL2* was significantly elevated when compared to the 12:12LE group. Furthermore, *TGF-B1* mRNA levels are reported to peak at 2 and 12 weeks post-myocardial damage in rats^34^. Similarly, a study of *P. obesus* which were also exposed to short photoperiods and HE diets showed an increase in *TGF-B1* levels in fat tissue at 10 weeks^25^. This suggests the time points of this study were unable to capture changes in inflammatory and pro-fibrotic markers.

Previous studies in *P. obesus* have found exposure to HE diet and/or circadian disruption increase myocyte hypertrophy^21,22,24^. Studies in rats and mice fed high fat diets have reported similar findings^30,32^. Consistent with this, the current study found that after 8-weeks there were increases in the hypertrophy genes *Gata4*, *Merf2C* and *Nppa* in the *P. obesus* exposed to the HE diet and short photoperiod. Despite this, we did not witness evidence of cardiomyocyte hypertrophy using histological analyses, suggesting that elevation in hypertrophy genes precedes physical increases cardiomyocyte size. In the 20-week cohort of *P. obesus* there was a reduction in cardiomyocyte size in the 5:19HE group, when compared to LE diet and/or normo-photoperiod (12:12). This was unable to be explained by reductions in hypertrophy gene expression as they remained unchanged. The most significant variation from previous studies is the length of time on HE diet and short photoperiod (16 weeks *vs.* 20 weeks), which in turn prolongs the overall period of abnormal glucose control in these animals. Stem cell recruitment to the myocardium, their survival and proliferation as part of the myocardial repair process are impaired by hyperglycaemia^35,36^. Extended abnormal glucose tolerance, as is the case for the 20-week group in this study, may tip the balance from an inflammatory-driven hypertrophy response into myocardial atrophy due to diminished stem cell recruitment/repair.

*Per2* is one of the core clock genes that is used to determine circadian rhythmicity of the clock molecular machinery that is present in every cell. In the 20-week cohort, *Per2* levels were significantly lower in the 5:19HE group, when compared to the 12:12HE group. Interestingly, the 5:19HE group also had pronounced myocardial perivascular fibrosis. Our findings of a decline in *Per2* are consistent with previous studies that found deletion of *Per2* in rodent models induces deleterious cardiovascular consequences^5,37,38^. The daily average of *Per2* gene expression did not change after 8 weeks, although there were alterations in the daily rhythm in *Per2* gene expression that were diminished in the 5:19HE group. This response was mimicked by the inflammatory genes *Rela* and *CCl2* and fibrotic gene *Tgfb1,* in which the daily circadian rhythm of their expression was abolished in the 5:19HE group. *CCl2* is controlled by the core clock protein Bmal1^39^, therefore diminished rhythms in *Per2* will result in diminished rhythms in all clock-controlled genes, such as *CCl2,* consistent with the observations of this study.

In conclusion, using the well-characterised *Psammomys obesus* model of T2DM we simulated circadian disruption, similar to a shift worker, combined with a HE diet to assess the onset of features of T2DM and myocardial pathophysiology. We show that disrupted circadian cycles cause impaired glucose tolerance. Furthermore, fasting glucose levels were significantly increased with HE diet exposure and/or circadian disruption after 8 weeks. This onset in the features of T2DM with circadian disruption is found to coincide with the presence of myocardial perivascular fibrosis and an increase in *Col1a1* expression. There were limited changes in inflammatory (*Rela, CCl2*) and pro-fibrotic (*Tgfb1*) genes, although their circadian rhythmicity was diminished with circadian disruption and HE diet. These findings provide insights into the potential adverse effects on the heart that may be experienced by shift workers due to T2DM that is induced by circadian dysfunction and amplified by a HE diet. Our studies may provide guidance for the prevention of diabetes-related cardiac fibrosis in shift workers in which a healthy diet and reduced time periods of circadian disruption should be promoted as a strategy for preventing cardiac dysfunction.

## Methods

### Animal experiments

This study was carried out in compliance with the ARRIVE guidelines. HsdHu diabetes-prone male sand rats (*P. obesus*, 6–7 months old) were initially maintained on a low energy diet (Koffolk Ltd, Israel) before assignment to experimental groups. All experimental procedures conformed to the NIH guidelines for the care and use of laboratory animals and were approved by the Institutional Animal Care and Use Committee (IACUC) of Tel Aviv University (#L15055). Adult male sand rats (n = 11–15/group) were exposed to the following conditions: (1) normal photoperiod, low energy diet (12:12LE); (2) normal photoperiod, high energy diet (12:12HE); (3) short photoperiod, low energy diet (5:19LE) and (4) short photoperiod, high energy diet (5:19HE). Animals in the LE groups were fed a special low-energy diet (14% protein, 1.7% fat, 15.4% Crude fibre; Product No.: 1078; Koffolk Ltd., Israel), which is the caloric equivalent to their native diet. Animals in the HE groups were fed the standard rodent diet (21% protein, 4% fat, 4% Crude fibre; Product No.: 2018; Koffolk Ltd.), which contains a higher caloric density and known to drive T2DM development in *P. obesus*^20^. In week 10, sand rats were weighed and then euthanized at four different time points post- ‘lights on’ (ZT0), followed by ZT2 and then at 6 h intervals of ZT8, ZT14, ZT20. When sacrificing the animals during the dark hours (ZT14 and ZT20), the head of the sand rat was covered with aluminium foil until the brain was excised to prevent exposure to light. Hearts were excised, weighed and snap frozen for histological and RT-qPCR analyses.

In a parallel cohort (n = 12/group), *P. obesus* were maintained with the same diet and photoperiod exposure conditions for a longer time period of 20 weeks. Euthanasia was conducted two hours following ‘lights on’ (ZT 2) and their hearts were excised, weighed and snap frozen for histological and RT-qPCR analyses.

All animals had fasting glucose concentrations assessed using a glucometer. Oral glucose tolerance tests (OGTT) were performed in animals fasted for 4 h. Blood was collected from the tip of the tail at time 0 and 120 min^21^. Tests were performed at ZT 2 by administering 2 g glucose/kg body weight dissolved in 1 ml water using gastric gavages, inserted through the mouth into the stomach.

### Histological analysis

The apex of snap-frozen hearts were formalin fixed and paraffin embedded then sectioned on a microtome at a thickness of 5 µm. Two sections were taken ~ 50 µm apart and stained with Masson’s Trichrome (Sigma). Heart vessels were chosen randomly within the heart sections. An individual vessel was then photographed under × 400 magnification, with five photos per section selected on two sections (n = 10 images per animal). Perivascular cardiac fibrosis was determined by calculating a ratio for the area of collagen (blue) surrounding the vessels divided by the vessel area. Similarly, two sections were taken ~ 50 µm apart and stained with Haemotoxylin and Eosin (Fronine, Thermofisher) then imaged at two separate locations within the heart (total 4 images per animal) to define cell morphology. To determine cardiomyocyte size, the area was calculated by dividing the area of the Eosin stain (Cytoplasm) by the number of haematoxylin stained nuclei. We focussed our analyses on regions of cardiomyocytes that had centralized nuclei. All images were taken using an AxioLab microscope attached to a camera (Zeiss) then analysed with Image-Pro Premier 9.2 (64 bit).

### Gene expression analysis

Total RNA was extracted from the apex region of snap-frozen myocardial tissue using TRI-reagent (Sigma). RNA was quantified and quality assessed by spectrophotometric analysis (Nanodrop). Quantitative real-time PCR was performed using previously published primers to detect expression of pro-alpha1 chains of collagen type 1 (*Col1a1*), Period 2 (*Per2*), C–C motif chemokine ligand 2 (*Ccl2*), transforming growth factor-β1 (*Tgfb1*), Bcl2 associated X protein (*Bax*), sarco(endo)plasmic reticulum Ca2+ ATPase 2 (*Serca2*) with housekeeping gene *Cyclophilin*^24,40^. Primers for p65 subunit of nuclear factor kappa B (*p65 NF-κB*, *Rela*) [F: 5′-ATG GAC GAT CTG TTT CCC CTC-3′, R: 5′-CAC TTG TAG CGG AAC GCA T-3′], B-cell lymphoma 2 (*Bcl2*) [F: 5′-AGC ATG CGA CCT CTG TTT GA-3′, R: 5′-TCA CTT GTG GCC CAG GTA TG-3′], *Gata4* [F: 5′-GCG CCG TAA TGC GAG G-3′, R: 5′-TCT TGG GCT TCC GTT TTC TGG-3′], *Mef2C* [F: 5′-TTA CCA GGA CAA GGA ATG GGA G-3′, R: 5′-CCA GCC AGT TAC GAG CC-3′], *Nppa* [F: 5′-CTA GAC CAC CTG GAG GAG AAG A-3′, R: 5′-AGA GCA CCT CCA TCT CTC TGA GT-3′], *Myh6* [F: 5′-CAA CCT CAA TGA TGC GAG GA-3′, R: 5′-CTT CCA GCT TGC GCT TCT TG-3′] and *Myh7* [F: 5′-CCC GAG GCA AGC TCA CTA TAC-3′, R: 5′-CAC CTC TGA GTT GGC CTT GG-3′] were designed by aligning *Homo sapiens*, *Mus musculus* and *Rattus norvegicus* sequences and generated based on the overlapping consensus sequences using Primer Blast. Relative changes in average gene expression were determined across the 4 groups irrespective of the time point of euthanasia and normalized using the ^ΔΔ^*Ct* method to *Cyclophilin* and to the 12:12LE control group. For the 8-week cohort, animals within each group were euthanized at 6-h intervals from ZT0 (lights on). Daily rhythmicity of gene expression was determined by measuring genes at different time points post-ZT0 (i.e. ZT2, ZT8, ZT14 and ZT20) and normalized using the ^ΔΔ^*Ct* method to *Cyclophilin* and the 12:12LE group euthanized at ZT2.

### Statistical analysis

Histology data was expressed as Mean ± SEM. Gene expression data was expressed as either averaged daily levels (Mean ± SEM, n = 11–15/group) or daily rhythms (Mean ± SD, n = 2–6/group). Differences between treatment groups or time points within groups were calculated using a two-way ANOVA with Tukey’s multiple comparisons post hoc test. Significance was set at a two-sided *P* < 0.05.
